# Impact of copper deficiency in cows on immune system function in offspring: a study using lymphocyte immunophenotyping

**DOI:** 10.2478/jvetres-2026-0040

**Published:** 2026-08-12

**Authors:** Beata Abramowicz, Łukasz Kurek, Urszula Lisiecka, Katarzyna Żarczyńska, Anna Szczotka-Bochniarz

**Affiliations:** 1 Department and Clinic of Animal Internal Diseases, Department of Epizootiology and Clinic of Infectious Diseases, University of Life Sciences in Lublin, 20-950 Lublin, Poland; 2 Department of Epizootiology and Clinic of Infectious Diseases, University of Life Sciences in Lublin, 20-950 Lublin, Poland; 3 Department of Internal Medicine with Clinic, University of Warmia and Mazury in Olsztyn, 10-719 Olsztyn, Poland; 4 Department of Bacteriology and Bacterial Animal Diseases, National Veterinary Research Institute, 24-100 Puławy, Poland

**Keywords:** immunity, flow cytometry, hypocupraemia, lymphocyte subpopulations, maternal nutrition

## Abstract

**Introduction:**

Copper (Cu) deficiency is a common, often subclinical problem in dairy cattle herds that can adversely affect haematopoietic processes and immune function. This study aimed to evaluate the effect of hypocupraemia in cows on selected haematological parameters and identify the immunophenotype of calf lymphocytes.

**Material and Methods:**

The study included Holstein–Friesian cows and their calves aged two to six weeks, which were divided into two groups: a hypocupraemic group (17 cows and 17 calves) and a normocupraemic group (18 cows and 18 calves). Haematological and biochemical analyses were performed, and lymphocyte subpopulations (CD4^+^, CD8^+^, CD21^+^ and major histocompatibility complex (MHC) class II^+^ were determined by flow cytometry.

**Results:**

Cows with hypocupraemia exhibited reduced HGB and HCT values. Calves born to cows with Cu deficiency showed decreased erythrocyte indices, a reduced CD4^+^:CD8^+^ ratio and a lower percentage of CD21^+^ B lymphocytes and MHC class II^+^ lymphocytes, indicating impaired immune function. In these calves, the CD4^+^:CD8^+^ ratio was significantly lower compared to healthy cows and calves, suggesting an imbalance in T-lymphocyte subpopulations.

**Conclusion:**

The obtained results indicate that even moderate copper deficiency in pregnant cows may negatively influence the haematological and immunological status of their offspring. These findings underline the necessity of systematic monitoring of copper status in dairy herds in order to prevent subclinical deficiencies and their long-term consequences for calf health and immunity.

## Introduction

In dairy cattle, mineral deficiencies are a significant and current health and production problem. Historically, macroelement deficiencies were predominant, but nowadays, microelement deficiencies are increasingly observed, particularly those related to copper. Despite the growing awareness of breeders with regard to balancing energy and minerals in a feed ration, the issue of adequate microelement supply continues to be marginalised in dairy cattle feeding. According to the recommendations of the US National Academies of Science, Engineering and Medicine, (21), cows’ requirement for Cu is 12 mg kg^−1^ dry matter during the dry period, and 11 mg kg^−1^ dry matter during lactation. However, a significant proportion of the mineral and vitamin supplements used contain insufficient amounts of this element, or do not contain it at all.

Copper deficiency (hypocupraemia) in cattle is often diagnosed only when clear clinical symptoms become apparent, including characteristic depigmentation of the hair around the eyes (“copper glasses”), or chronic diarrhoea that is difficult to treat. Long-term, mild hypocupraemia is more often manifested as non-specific disorders, such as periodic decreases in appetite, reduced milk yield, anaemia or changes around the hooves, with a lack of classic symptoms. The disorders described may already occur at blood copper concentrations within the lower limits of reference ranges and often respond well to supplementation. The effectiveness of compensating for copper deficiency is, however, determined by many factors, including the presence of antagonists in the diet (*e.g*. iron, molybdenum or sulphur) and the functional state of the liver, which is responsible for the synthesis of ceruloplasmin (16, 18).

The proper functioning of numerous metabolic processes depends on copper sufficiency. Copper deficiency impairs the activity of phagocytic cells such as neutrophils and macrophages, reducing the body’s ability to combat pathogens. It has also been shown that hypocupraemia affects elements of adaptive immunity, including T and B lymphocyte populations, and the balance between CD4^+^ and CD8^+^ T lymphocyte subpopulations, the disturbances of which may indicate impaired immunoregulation (4, 6, 20).

In cattle breeding practice, attention is increasingly being paid to the relationship between the mineral status of cows and the health status of their calves. Clinical observations indicate that calves born to cows with clinical or subclinical copper deficiency exhibit reduced immunity. This is manifested by increased susceptibility to diarrhoea and respiratory diseases, and slower growth rates, despite the absence of clear symptoms of Cu deficiency and serum concentrations within the lower limits of the reference ranges (11, 15, 22).

The aim of this study was to assess the effect of copper deficiency in cows during gestation on the immunity of their calves. The immune function of calves born to cows with copper deficiency was assessed using lymphocyte immunophenotyping, which enables detailed analysis of selected immune cell populations.

## Material and Methods

The study was conducted on 70 animals on two dairy cattle farms located in southern Poland. The study included 35 Holstein–Friesian cows aged three to six years, with body condition scores of 3/5, and their offspring (n = 35) aged three to six weeks. The animals were divided into two experimental groups based on anamnesis, clinical examination and serum copper concentration test results. Copper concentration tests were performed two weeks before parturition, in accordance with the herd-monitoring protocols on these farms. Within each group, two subgroups were established: subgroups Ia and IIa in the cows and subgroups Ib and IIb in the calves. Subgroup Ia comprised 18 clinically healthy cows from a herd without diagnosed copper deficiency, in which a normal course of pregnancy and calving was confirmed by an anamnesis obtained from the herd owner. The average copper concentration in the blood serum was 13.35 ± 1.50 μmol/L. Subgroup Ib consisted of 18 clinically healthy calves with no reported health problems, and no abnormalities observed during clinical examination. The average copper concentration in the blood serum of calves in this group was 13.45 ± 0.66 μmol/L. Subgroup IIa consisted of 17 cows with hypocupraemia originating from a herd under monitoring, in which serum copper concentrations ranged from 7.5 to 11 μmol/L. The variation in Cu levels was associated with the current physiological stage and milk yield of the animals. It should be emphasised that despite the presence of hypocupraemia, the farmer did not implement targeted mineral supplementation or provide mineral licks because there were no clear clinical signs. According to the medical history, animals in this group showed periodic reductions in feed intake and decreased milk production. The clinical examination showed paleness of the mucous membranes, varying degrees of dyspnoea and changes in the form of redness and swelling in the interdigital space, whereas six cows exhibited characteristic periocular hair depigmentation associated with copper deficiency, as well as greying of the hair in the head and neck region. The average copper concentration in the blood serum of cows in this group was 10.25 ± 1.18 μmol/L. Subgroup IIb comprised 17 calves born to cows with hypocupraemia. These animals showed increased susceptibility to infectious agents, as evidenced by episodes of diarrhoea and/or respiratory disease. At parturition, most of the calves had an average birth weight approximately 10% lower than that of calves in subgroup Ib. The average copper concentration in the blood serum of calves in this group was 11.83 ± 1.68 μmol/L.

The farms implemented a total mixed ration feeding system, and the feed ration consisted of maize silage, maize grain, hay silage, grass silage, hay, straw, pelleted feed, farm-produced cereals, feed additives with a protein content of 18 to 24%, premix and a mineral and vitamin supplement. The feed rations were designed based on milk yield, the current physiological period and cow body weight. Calves were fed milk replacer formulated for their age and weight.

Blood tests in the cow group were conducted in accordance with the rules for monitoring dairy cattle herds at the Clinic of Animal Internal Diseases of the University of Life Sciences in Lublin. Samples were taken during or after milking, and before the first total mixed ration feeding in the morning, approximately two weeks before parturition. Blood was also collected from the calves prior to the first morning feeding. It was collected from the external jugular vein using a vacuum blood collection kit consisting of a 20G needle (0.9 × 38 mm) and a holder (BD Vacutainer, Becton Dickinson, Franklin Lakes, NJ, USA). To obtain serum, blood was collected into tubes containing a clot activator (9 mL, Vacuette, Greiner Bio-One, Kremsmünster, Austria), whereas to obtain whole blood, blood was collected into tubes containing K3EDTA (2 mL, Vacuette, Greiner Bio-One). The blood was centrifuged for 15 min at 1,500 rpm. The concentrations of copper and iron in the serum were determined by atomic absorption spectrometry with excitation in an acetylene–air flame. The morphological examination determined the following: WBC, RBC, HGB, HCT, MCV, MCH, MCHC and PLT using a Horiba scil Vet abc Plus automatic analyser (Horiba, Montpellier, France). Manual smears were performed on glass microscope slides. The preparations were stained by the May–Grünwald–Giemza method using a MYTHIC TS haematological smear staining apparatus (Orphée, Geneva, Switzerland), and the percentages of leucocyte subpopulations, *i.e*. band neutrophils, segmented neutrophils, lymphocytes and monocytes, were assessed.

The immunophenotype of lymphocytes was determined using a BD FACSVerse flow cytometer (BD Biosciences, Franklin Lakes, NJ, USA). The experiment used antibodies against cell differentiation antigens (Bio-Rad, Hercules, CA, USA): anti-CD4 labelled with Alexa Fluor647 dye, anti-CD8 labelled with FITC, anti-CD21 labelled with FITC and anti–major histocompatibility complex (MHC) class II labelled with R-phycoerythrin dye. The study used the direct labelling method. Whole blood samples (50 μL) were incubated with an appropriate amount of antibody for 20 min in the dark at room temperature. This was followed by a 20-min incubation of the samples with 1 mL of ammonium chloride solution to lyse the red blood cells. The final stage of the test was analysing the samples using a flow cytometer. In each test, 10,000 leucocytes were analysed. Leucocyte subpopulations were gated based on their size and granularity, using forward-scatter (FSC) and side-scatter (SSC) parameters. The intensity of antibody labelling was analysed on fluorescence and SSC plots (Fig. 1). The results were expressed as the percentage of positive cells within the gated lymphocyte population and as the CD4^+^:CD8^+^ (T-helper: T-cytotoxic cells) ratio. To optimise gating, appropriate controls were used and analysed under the same conditions as the experimental samples. BD FACSuite CS&T (cytometer setup and tracking) Research Beads (BD Biosciences, San Jose, CA, USA) and BD Calibrite 3 Beads (BD Biosciences, San Jose, CA, USA) were used for calibration and validation procedures. Analyses for each antibody were carried out using the same protocols, instrument settings and voltages (FSC 233.5 V, SSC 354.8 V, FITC 501 V, phycoerythrin 473.1 V and Alexa Fluor647 544.6 V). All blood samples were tested at a low flow rate. The flow cytometry results were corroborated by haematological tests and microscopic evaluation of leucocytes.

**Fig. 1.**
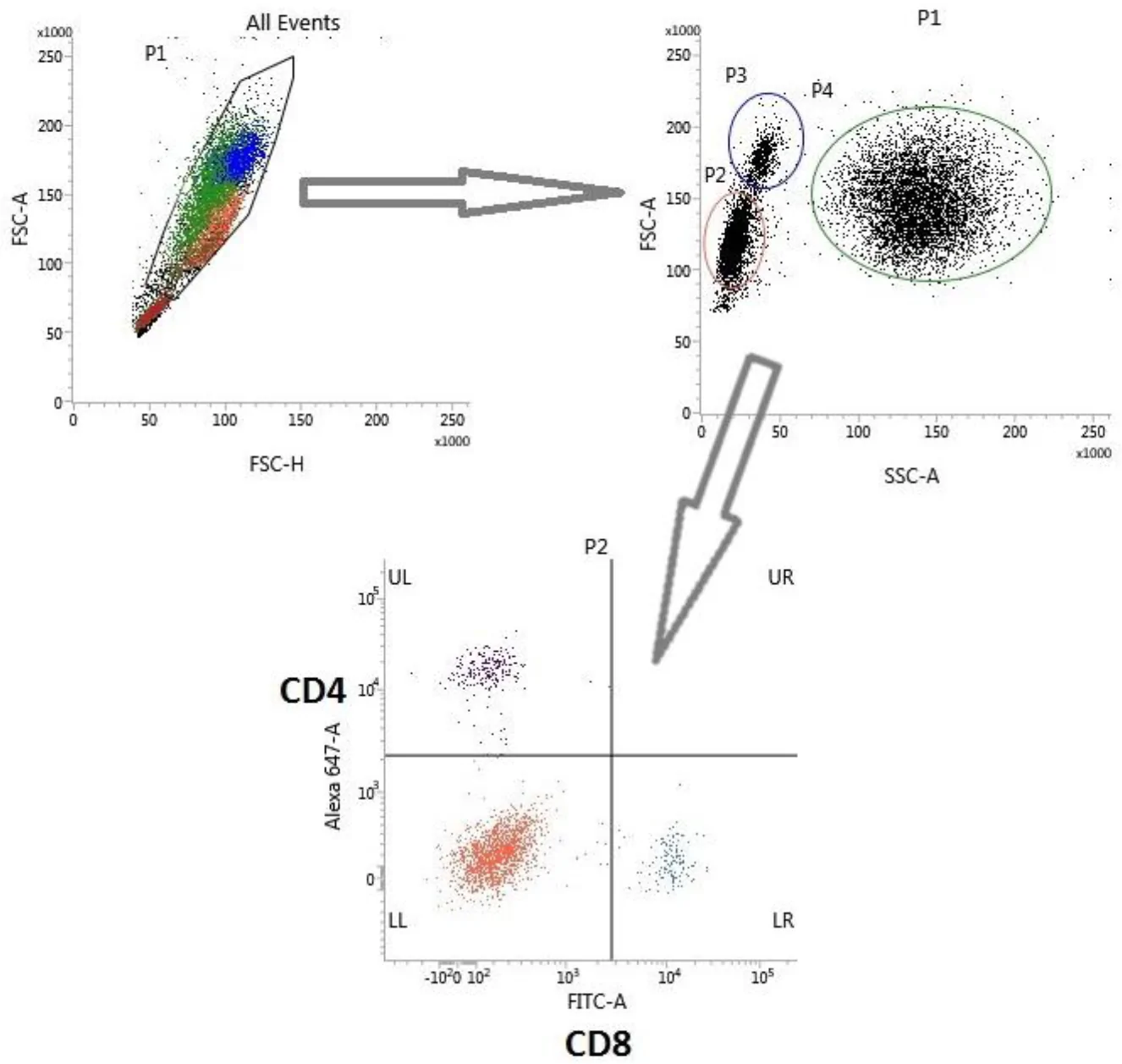
Gating strategy for bovine lymphocyte immunophenotyping. Single cells were selected in a forward-scatter (FSC) pulse area (A) *vs* FSC pulse height (H) plot. Lymphocytes were identified/gated in an FSC-A *vs* side-scatter (SSC)-A plot based on their size and granularity, and their fluorescences were gated in SSC-A to fluorescence histograms

The results of haematological, immunophenotypic and biochemical tests were statistically analysed using the Mann–Whitney rank test. The calculations were performed at significance levels of α = 0.05.

## Results

Tables 1 and 2 present the results of haematological, biochemical and immunophenotypic tests of normocupraemic cows, hypocupraemic cows, calves born to normocupraemic mothers and calves born to hypocupraemic mothers.

**Table 1.** Results of biochemical and haematological tests on cows and calves

Parameter	Reference range (19)	Normocupraemic cows, subgroup Ia	Normocupraemic calves, subgroup Ib	Hypocupraemic cows, subgroup IIa	Hypocupraemic calves, subgroup IIb
Cu (μmol/L)	12–20	13.35 ± 1.50^a^	13.45 ± 0.66^a^	10.25 ± 1.18^b^	11.83 ± 1.68^ab^
Fe (μmol/L)	21.5–35.8	27.50 ± 1.33^a^	23.70 ± 1.66^b^	31.90 ± 3.98^a^	25.25 ± 4.33^ab^
WBC ×10^9^/L	6.25–9.5	9.50 ± 3.89^a^	10.03 ± 1.34^a^	6.60 ± 1.61^b^	9.70 ± 3.97^a^
GP (%)	0–2	3.00 ± 1.00	1.50 ± 0.71	3.67 ± 3.79	2.00 ± 0.00
GS (%)	23–37	44.50 ± 15.80	31.50 ± 15.59	39.50 ± 10.85	35.67 ± 24.54
L (%)	53–67	49.00 ± 13.34	64.00 ± 15.01	52.25 ± 9.64	60.67 ± 21.46
M (%)	0–4	2.00 ± 0.00	2.00 ± 0.00	0.00 ± 0.00	0.00 ± 0.00
RBC (×10^12^/L)	5.0–7.0	7.23 ± 0.46^a^	9.24 ± 0.93^b^	7.08 ± 0.57^a^	8.50 ± 1.91^ab^
HGB (g/L)	105–140	114 ± 1.83^a^	110.00 ± 1.83^a^	101.75 ± 8.22^b^	101.00 ± 1.84^b^
HCT (L/L)	0.3–0.4	0.35 ± 0.01^a^	0.31 ± 0.02^ab^	0.29 ± 0.02^b^	0.29 ± 0.07^b^
MCV (fL)	40–60	48.75 ± 2.99^a^	36.00 ± 3.46^ab^	46.00 ± 6.98^ab^	36.00 ± 1.73^b^
MCH (fmol/L)	0.9–1.5	0.98 ± 0.06^a^	0.71 ± 0.05^b^	0.87 ± 0.10^c^	0.70 ± 0.03^b^
MCHC (mmol/L)	16–21	20.06 ± 0.31	19.81 ± 0.62	19.40 ± 0.22	19.59 ± 0.57
PLT (×10^9^/L)	200–800	635 ± 164.04^a^	807.25 ± 253.80^b^	856.00 ± 83.63^b^	815.00 ± 145.66^b^

1 GP – granulocytes; GS – segmented granulocytes; L – lymphocytes; M – monocytes; concentrations are given as means ± SD;

a, b and c – values in a row with different superscript letters differ significantly at α = 0.05

**Table 2.** Lymphocyte immunophenotypes in cow and calf serum

	Normocupraemic cows, subgroup Ia	Normocupraemic calves, subgroup Ib	Hypocupraemic cows, subgroup IIa	Hypocupraemic calves, subgroup IIb
TCD8%	8.99 ± 0.72^a^	7.45 ± 0.12^a^	13.35 ± 2.61^b^	13.18 ± 4.73^b^
TCD4%	23.19 ± 2.85^a^	16.12 ± 1.54^b^	27.60 ± 5.03^ac^	19.31 ± 3.91^b^
CD4^+^:CD8^+^ ratio	2.58 ± 0.23^a^	2.16 ± 0.17^a^	2.14 ± 0.56^a^	1.54 ± 0.36^b^
BCD21%	26.35 ± 2.04^a^	26.5 ± 4.72^a^	24.88 ± 6.27^a^	17.01 ± 14.30^b^
MHC II%	45.12 ± 4.38^a^	45.35 ± 16.99^a^	36.15 ± 5.87^b^	29.74 ± 16.20^b^

1 TCD4^+^ – CD4 T helper cells; TCD8^+^ – CD8 T helper cells; MHC II – major histocompatibility complex class II; BCD21 – CD21 B cells; values are given as means ± SD;

a, b and c – values in a row with different superscript letters differ significantly at α = 0.05

In cows with hypocupraemia, copper concentrations were significantly lower than in healthy cows. A similar trend was observed in calves born to cows with hypocupraemia, with the average Cu concentration being significantly lower than that in calves born to cows without deficiencies. The Cu concentrations in this group of animals (cows and calves) were also below the reference values reported by Meyer and Harvey (19). However, the concentrations of this microelement fell within the normal range in clinically healthy animals. The average iron concentrations in the groups studied were within the adopted reference values (19).

Analysis of leucocyte parameters showed that in cows with hypocupraemia, leucocyte counts were significantly lower than in healthy cows. In calves born to mothers with Cu deficiency, the WBC counts were similar to those observed in healthy calves, but exhibited greater variability. Blood smears showed higher percentages of granulocytes in cows with hypocupraemia, accompanied by lower percentages of lymphocytes compared with healthy cows. A similar trend, although less pronounced, was observed in calves born to mothers with hypocupraemia. These values fell within the adopted reference ranges (19).

Lymphocyte immunophenotyping revealed significant differences between the groups. Cows with hypocupraemia showed higher average percentages of CD8^+^ and CD4^+^ T lymphocytes, but the CD4^+^:CD8^+^ ratio was lower than that in healthy cows. Calves born to cows with hypocupraemia exhibited a statistically significant decrease in the average CD4^+^:CD8^+^ ratio compared to those in healthy cows and calves, indicating an imbalance in T-lymphocyte subpopulations. In addition, these calves had lower percentages of CD21^+^ B lymphocytes and MHC class II^+^ lymphocytes than calves born to healthy mothers. Representative results of flow cytometric immunophenotyping are presented in Fig. 2.

**Fig. 2.**
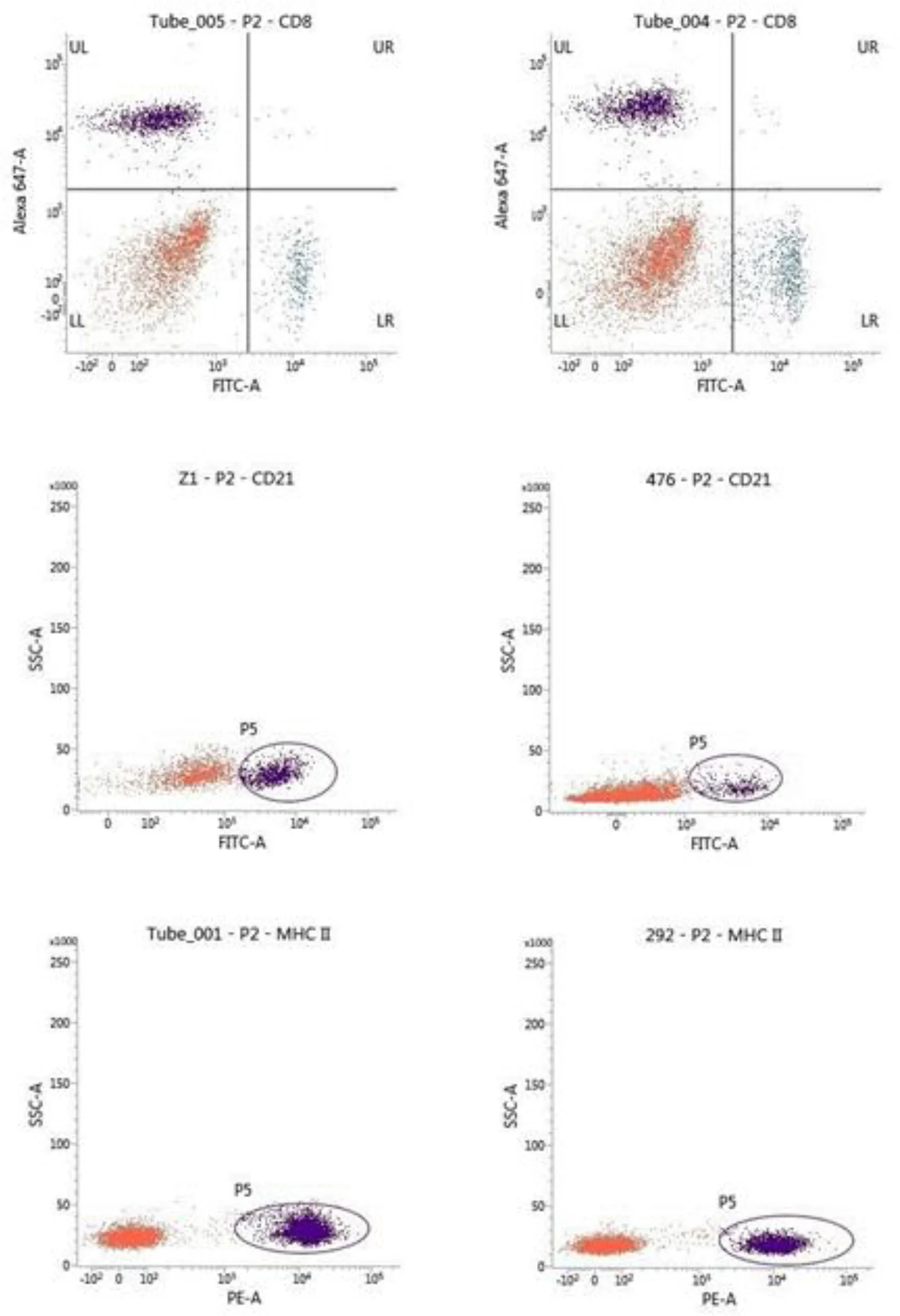
Representative flow cytometric dot plots of peripheral blood lymphocytes in cows with hypocupraemia and healthy control animals. A – healthy cows; B – cows with hypocupraemia. SSC – side scatter; A – pulse area; MHC II – major histocompatibility complex class II; PE – phycoerythrin

In terms of RBC parameters, cows with hypocupraemia had lower HGB, HCT and MCH values than healthy cows. Calves born to cows with hypocupraemia showed lower HGB and HCT values than healthy calves. The erythrocyte counts in calves were higher than the counts in cows, regardless of group, but were accompanied by a lower MCV and MCH amount.

The PLT count was higher in cows with hypocupraemia than in healthy cows. In calves, regardless of their mothers’ status, PLT values exceeded the upper limit of the normal range (19), with the highest values observed in calves born to cows with hypocupraemia.

## Discussion

The present results confirm that moderate hypocupraemia significantly affects haematological and immunological parameters in cows and their offspring. In contrast to macroelement deficiencies, Cu deficiencies are often subclinical and remain undiagnosed until clinical symptoms appear or the animals’ production and health parameters deteriorate (16, 18). These findings indicate that even moderate long-term hypocupraemia in cows can significantly affect haematological and immunological parameters in both cows and their calves.

In the group of cows with hypocupraemia, a reduced copper concentration in blood serum (10.25 ± 1.18 μmol/L) was observed, accompanied by mildly pronounced clinical symptoms, including paleness of the mucous membranes, inflammatory changes within the interdigital spaces, and, in some animals, characteristic periocular hair depigmentation. These observations are in line with the reports by Abramowicz *et al*. (1, 2) and López-Alonso & Miranda (16), who indicated that in the course of long-term copper deficiency, the first clinical symptoms may include a periodic decrease in feed intake, a decrease in milk yield and signs of anaemia (paleness of the mucous membranes and dyspnoea), often appearing before the onset of typical changes in coat colour. This study ruled out the possibility that the observed paleness of the mucous membranes and signs of anaemia were due to primary iron deficiency. The haematological changes observed were exclusively associated with hypocupraemia, as confirmed by studies by other authors (7, 10), indicating the crucial role of copper in iron metabolism and the normal course of erythropoiesis in cattle.

Analysis of red blood cell parameters showed that both cows with hypocupraemia and their calves exhibited significant abnormalities in erythrocyte indices compared with healthy animals. Cows with copper deficiency were found to have reduced haemoglobin and haematocrit values, while calves born to these dams were also found to have reduced mean corpuscular volume and mean corpuscular haemoglobin values. These changes indicated a trend towards developing microcytic and hypochromic anaemia. These observations were consistent with previous reports, which stated that the reference MCH values for calves aged one to four weeks ranged from 10.99 to 14.62 pg, while those for calves aged five to eight weeks range from 10.51 to 12.81 pg (13). In the present study, MCH values in calves in the hypocupraemic subgroup were at the lower end of these ranges or below, confirming the occurrence of erythropoiesis disorders already in early life.

The observed haematological changes can be explained by pathophysiological mechanisms associated with copper deficiency. As demonstrated, Cu is an essential component of ceruloplasmin and hephaestin, *i.e*. enzymes that determine the mobilisation of iron from the liver and its transport and utilisation in the bone marrow. Copper deficiency impairs the activity of these proteins, reducing iron availability for erythropoiesis, even with normal or elevated serum Fe levels (5, 26). Consequently, microcytic and hypochromic anaemia develops, as confirmed by other authors (8, 12). The results obtained in calves born to cows with hypocupraemia are particularly significant. Although the average Cu concentration in calf blood serum fell within the reference value range at its lower end, these animals showed a clearly increased susceptibility to infectious diseases, slower foetal growth rates and significant changes in immunological parameters. This phenomenon is supported by reports in the literature indicating that the mineral status of the mother during gestation can affect the offspring’s immune function, even in the absence of evident Cu deficiency in calf serum (23, 24). In the present study, calves born to cows with hypocupraemia were found to have a reduced CD4^+^:CD8^+^ lymphocyte ratio and reduced percentages of CD21^+^ B lymphocytes and MHC class II^+^ cells. The literature indicates that the normal CD4^+^:CD8^+^ ratio in healthy cattle should exceed 1, and in Holstein–Friesian cows it often ranges from 2.3 to 2.4 (9, 17). The decrease in this ratio observed in the authors’ studies may indicate an imbalance in immunoregulation, contributing to the weakening of both cellular and humoral immunity. Copper has a crucial role in the functioning of immune cells, especially neutrophils and macrophages, influencing their phagocytic capacity and bactericidal activity (3, 6, 25). Copper deficiency impairs innate immune mechanisms, increasing susceptibility to infections, particularly during the perinatal period and early life of calves (14, 20). The results obtained confirm that calves born to mothers with copper deficiency represent a group at increased risk of infectious diseases, even if they do not show clinical signs of hypocupraemia.

## Conclusion

The results indicate that chronic, even moderate, hypocupraemia in cows during gestation adversely affects the development and function of calves’ immune systems. This phenomenon is of practical importance, as it can lead to an increased incidence of infectious diseases in calves and deterioration in rearing results. Based on the results, it is justified to include copper testing in every blood test panel for cows. The authors also believe that even a slight Cu deficiency should be corrected, given its impact on calf health and foetal weight. The findings of this study highlight the need for further research, particularly regarding the assessment of colostrum quality and the long-term impact of copper deficiency in mothers on the health status and productivity of their calves.
